# The MedSeq Project: a randomized trial of integrating whole genome sequencing into clinical medicine

**DOI:** 10.1186/1745-6215-15-85

**Published:** 2014-03-20

**Authors:** Jason L Vassy, Denise M Lautenbach, Heather M McLaughlin, Sek Won Kong, Kurt D Christensen, Joel Krier, Isaac S Kohane, Lindsay Z Feuerman, Jennifer Blumenthal-Barby, J Scott Roberts, Lisa Soleymani Lehmann, Carolyn Y Ho, Peter A Ubel, Calum A MacRae, Christine E Seidman, Michael F Murray, Amy L McGuire, Heidi L Rehm, Robert C Green

**Affiliations:** 1 Section of General Internal Medicine, VA Boston Healthcare System, 150 S Huntington Ave, Boston, MA 02130, USA; 2 Department of Medicine, Harvard Medical School, 150 South Huntington Avenue 152G, Boston, MA 02130, USA; 3 Division of Genetics, Department of Medicine, Brigham and Women’s Hospital, 41 Avenue Louis Pasteur, Suite 301, Boston, MA 02115, USA; 4 Laboratory for Molecular Medicine, Partners Healthcare Center for Personalized Genetic Medicine, 65 Landsdowne St., Cambridge, MA 02139, USA; 5 Department of Pathology, Brigham & Women’s Hospital and Harvard Medical School, 75 Francis St, Boston, MA 02115, USA; 6 Department of Medicine, Boston Children’s Hospital, 300 Longwood Ave, EN137, Boston, MA 02115, USA; 7 Department of Pediatrics, Harvard Medical School, 41 Avenue Louis Pasteur, Suite 301, Boston, MA 02115, USA; 8 Center for Biomedical Informatics & Department of Pediatrics, Harvard Medical School, 300 Longwood Ave, Enders-6, Boston, MA 02115, USA; 9 Center for Medical Ethics and Health Policy, Baylor College of Medicine, BCM-Jewish Institute for Research, Houston, TX 77030, USA; 10 Department of Health Behavior & Health Education at University of Michigan School of Public Health, 109 S. Observatory, SPH I Building, Room 3854, Ann Arbor, MI 48109, USA; 11 Center for Bioethics, Division of General Internal Medicine and Primary Care, Department of Medicine, Brigham and Women’s Hospital and Harvard Medical School, 1620 Tremont Street, Suite 03-002, Room BC3-2G, Boston, MA 02120, USA; 12 Division of Cardiovascular Medicine, Department of Medicine, Brigham and Women’s Hospital, 75 Francis St., Boston, MA 02115, USA; 13 Fuqua School of Business, Duke University, 100 Fuqua Drive, Durham, NC 27708, USA; 14 Department of Genetics, Harvard Medical School, NRB-room 256 77 Avenue Louis Pasteur, Boston, MA 02115, USA; 15 Genomic Medicine Institute, Geisinger Health System, 1000 East Mountain Drive, Wilkes Barre, PA 18711, USA; 16 Genomes2People and Division of Genetics, Department of Medicine, Brigham and Women’s Hospital, Broad Institute and Harvard Medical School, 41 Avenue Louis Pasteur, Suite 301, 02115 Boston, MA, USA

**Keywords:** Whole genome sequencing, Genome report, Genomic medicine, Translational genomics, Primary care, Cardiomyopathy genetics

## Abstract

**Background:**

Whole genome sequencing (WGS) is already being used in certain clinical and research settings, but its impact on patient well-being, health-care utilization, and clinical decision-making remains largely unstudied. It is also unknown how best to communicate sequencing results to physicians and patients to improve health. We describe the design of the MedSeq Project: the first randomized trials of WGS in clinical care.

**Methods/Design:**

This pair of randomized controlled trials compares WGS to standard of care in two clinical contexts: (a) disease-specific genomic medicine in a cardiomyopathy clinic and (b) general genomic medicine in primary care. We are recruiting 8 to 12 cardiologists, 8 to 12 primary care physicians, and approximately 200 of their patients. Patient participants in both the cardiology and primary care trials are randomly assigned to receive a family history assessment with or without WGS. Our laboratory delivers a genome report to physician participants that balances the needs to enhance understandability of genomic information and to convey its complexity. We provide an educational curriculum for physician participants and offer them a hotline to genetics professionals for guidance in interpreting and managing their patients’ genome reports. Using varied data sources, including surveys, semi-structured interviews, and review of clinical data, we measure the attitudes, behaviors and outcomes of physician and patient participants at multiple time points before and after the disclosure of these results.

**Discussion:**

The impact of emerging sequencing technologies on patient care is unclear. We have designed a process of interpreting WGS results and delivering them to physicians in a way that anticipates how we envision genomic medicine will evolve in the near future. That is, our WGS report provides clinically relevant information while communicating the complexity and uncertainty of WGS results to physicians and, through physicians, to their patients. This project will not only illuminate the impact of integrating genomic medicine into the clinical care of patients but also inform the design of future studies.

**Trial registration:**

ClinicalTrials.gov identifier NCT01736566

## Background

The sequencing of the human genome has brought with it the promise of a genomic revolution for clinical medicine. Many already envision a time when each person’s genome will be sequenced and available over the course of the life span as a resource, providing guidance for personalized approaches to health maintenance and disease prevention and treatment. Whole genome sequencing (WGS) is the laboratory process of determining most, if not all, of the 3 billion DNA base pairs across the 46 chromosomes of an individual’s genome. The first human genome sequence in the year 2003 cost almost $3 billion and took more than 10 years to complete [1]. The costs of sequencing have dropped significantly since then, and patients and physicians increasingly have access to WGS services in research and clinical settings [2-10]. At the same time, thousands of new genetic associations with human disease have been identified [11]. WGS can capture much of this information in a single clinical test for an individual, thereby simultaneously delivering genetic results for rare Mendelian diseases, common polygenic diseases, and personalized pharmacogenomics-based medication safety and efficacy.

The potential benefits of WGS seem substantial for a health-care environment that is increasingly emphasizing a more patient-centered, personalized, and preventative approach to wellness. Genetically personalized strategies might counteract the patient and physician frustrations that sometimes stem from the one-size-fits-all paradigm of evidence-based medicine [12]. However, certain factors may obstruct the successful integration of WGS into clinical care. First, laboratories must develop scalable pipelines to sequence genomes, ensure the quality of WGS data, define the validity and utility criteria that variants should meet to be reported to physicians, and appropriately interpret and deliver WGS results to physicians and their patients [9]. Then, patients will look to their health-care providers for guidance on how to interpret and act on WGS information [13]. In the absence of evidence for the clinical validity and utility of WGS in most clinical settings, it is unknown how providers will use WGS results in clinical care. Without adequate physician preparedness, the introduction of such inherently complex and probabilistic risk information to the patient-physician encounter may result in clinical chaos. The application of WGS to large numbers of individuals thus has the potential to uncover unanticipated findings whose impact on clinical care is, at present, impossible to quantify. The resulting confusion, coupled with the instincts of patients and clinicians to order additional medical tests, has the potential to increase health-care costs and iatrogenic harm without increasing value [14,15].

Nevertheless, the application of sequencing to an individual’s health care is highly likely in one form or another. Patients have expressed the desire to integrate genome-wide information into the physician-patient relationship and may even feel that physicians have an obligation to do so [13]. The development of standards and procedures for the use of WGS information in clinical medicine is thus an urgent need [16], and yet there is insufficient evidence and considerable uncertainty in how to do so [17,18]. With this state of the science in mind, we are conducting the MedSeq Project: a feasibility study implementing two randomized trials of WGS in clinical medicine. We have designed a study protocol to enroll both physicians and their patients as study participants, to sequence and interpret patients’ genomes, and to deliver clinical genome reports to physicians for use in clinical care. In this study, the questions we seek to answer include the following:

How should a clinical molecular genetics laboratory process and report WGS results to physicians and their patients in an intelligible way without oversimplifying the inherent complexity and uncertainty of WGS data?

With education and appropriate support, will non-geneticist physicians feel adequately prepared to discuss and manage WGS results with their patients?

How will the delivery of WGS results, some with unclear clinical significance, impact the actions, attitudes and outcomes of patients and their physicians?

Below we describe the design of the MedSeq Project protocol. In particular, we discuss the rationale for our study design and describe our protocol for recruiting physicians and patients to the study, randomly assigning patient participants to receive WGS or standard of care, educating physician participants about WGS, and measuring the impact of introducing WGS into clinical medicine.

## Methods/design

### Models of genomic medicine

Genome sequencing will be integrated into clinical care in many ways. It is already demonstrating clinical utility for the diagnosis and treatment of certain cancers [5,19] and rare diseases [2,3,20-22] and shows promise for use in infectious disease outbreaks [23-25] and fetal diagnosis in prenatal medicine [7,26]. The design of the MedSeq Project models two archetypal scenarios for how WGS could be integrated into clinical care. First, in situations in which a patient presents with a particular family history, symptom, or clinical syndrome, the genomic sequence may be specifically interrogated for a genetic cause for that particular presentation, a scenario we call *disease-specific genomic medicine*. In this scenario, analysis of the genome focuses on known or suspected variants in relevant disease-associated genes. For many genetic conditions, sequencing of candidate genes is already common practice, and the interrogation includes rigorous evaluation of novel variants that may have little or no prior exposure in the scientific literature or in available databases. At the same time, WGS may uncover incidental findings not related to the original indication for sequencing [27,28]. In a very different scenario in which the patient is generally healthy and does not have a family history suggestive of a genetic condition, the genome could be sequenced as a part of routine preventive medicine. We call this scenario *general genomic medicine*. Whereas disease-specific genomic medicine mirrors today’s practice in medical genetics of investigating the underlying genetic etiology of a clinical presentation, general genomic medicine is conceptually different from any genetics commonly practiced today. It more closely resembles current population-based preventive screening measures in clinical practice, such as newborn screening for metabolic disorders and adult screening for breast, cervical, and colorectal cancer. Among individuals without a specific indication for WGS, general genomic medicine examines the genomes for disease variants meeting an agreed-upon threshold for clinical relevance. Given the higher risk of false-positive test results in this generally healthy population, general genomic medicine requires higher standards of certainty and clinical significance. It also incorporates well-established pharmacogenomic associations so that clinicians can query a patient’s sequence for the likelihood of drug efficacy and safety when a new medication is prescribed. Moreover, carrier status results for recessive Mendelian traits such as Tay-Sachs disease and cystic fibrosis allow patients and their family members to recognize the presence of carrier states and to consider preconception screening or prenatal surveillance.

To model *disease-specific genomic medicine*, we are drawing on the expertise in the diagnosis, management, and molecular etiology of hypertrophic and dilated cardiomyopathy (HCM and DCM) among our study investigators at the Brigham and Women’s Hospital Cardiovascular Genetics Center. To answer the question of how WGS might impact the clinical care of HCM and DCM as compared with standard of care, we are enrolling cardiologists and their patients with HCM or DCM who have previously undergone or are preparing to undergo targeted genetic testing for a panel of genes known to be associated with cardiomyopathy. One example of such a standard genetic test is the Partners Laboratory for Molecular Medicine’s Pan Cardiomyopathy Panel, a targeted interrogation of 51 genes associated with conditions such as HCM, DCM, arrhythmogenic right ventricular cardiomyopathy, and left ventricular non-compaction [29]. To model *general genomic medicine*, we are recruiting primary care physicians (PCPs) and their generally healthy adult patients from the network of primary care practices at Brigham and Women’s Hospital, a network of more than 100 PCPs at 13 sites in the greater Boston area serving a diverse patient population of almost 100,000. This model of general genomic medicine uses WGS as an adjunct to routine preventive care in a population of patients without a specific indication for genetic testing.

### Overview of study design

**Figure 1:**
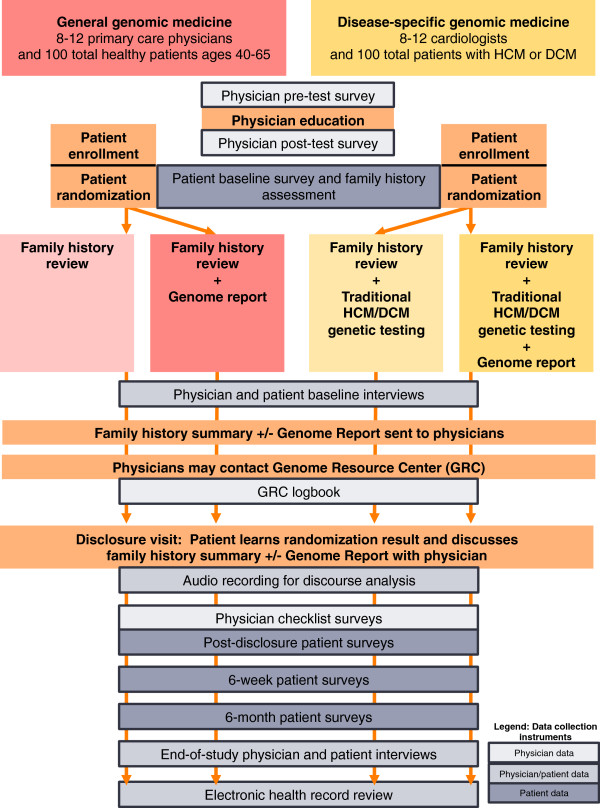
**MedSeq project study schema.** DCM, dilated cardiomyopathy; HCM, hypertrophic cardiomyopathy.

Figure 1 shows the MedSeq Project study schema. A randomized controlled trial design enhances our ability to isolate the effect of WGS disclosure on patient and physician attitudes and behaviors. Within each of the cardiology and primary care trials, we randomly assign patient participants to WGS versus standard of care and then study the impact of disclosing this risk information to physician and patient participants. The Partners Healthcare institutional review board approved this study protocol.

### Recruitment, enrollment, and sample size

We are recruiting a convenience sample of 8 to 12 PCPs and 8 to 12 cardiologists specializing in cardiomyopathy at our institution via individual and group e-mail communication and informational presentations for individual providers and group practices. Once enrolled in the study, each physician participant identifies and recruits 8 to 12 of his or her eligible patients, using study brochures, letters, phone calls, and in-person conversations. Potential patient participants are referred to study staff, who confirm eligibility and obtain informed consent during an in-person encounter. Our enrollment target is 200 total patient participants: 100 from primary care and 100 from cardiology. The MedSeq Project is a feasibility study that examines many outcomes; this targeted sample size is not formally designed to achieve statistical power for one specific primary outcome. Physician participants are compensated for their time at the end of the study regardless of the number of patients enrolled. Patient participants are compensated at the end of the study after completion of the 6-month survey and must complete all study surveys to receive compensation. A subset of patient participants is invited to complete qualitative interviews and receives additional compensation. Patient and physician incentives are intended to minimize losses to follow-up and occurrences of missing data.

### Patient inclusion and exclusion criteria

Table 1 shows the inclusion and exclusion criteria of the MedSeq Project patient participants. All patient participants must be receiving care from one of the physician participants, all of whom are affiliated with our institution. Patient participants in the primary care trial are generally healthy adults who are 40 to 65 years old. Patient participants in the cardiology trial have a diagnosis of HCM or DCM and have prior or concurrent targeted genetic testing for this condition. Any patient with a score of more than 14 or more than 16 on the anxiety and depression subscales, respectively, of the Hospital Anxiety and Depression Scale (HADS) [30] is excluded from enrollment; a study clinician explains the HADS result to the patient and makes appropriate clinical referrals, as necessary.

**Table 1:** Overall inclusion and exclusion criteria of the MedSeq project patient participants, plus additional criteria specific to the primary care or subspecialty cardiology trials

	**Both trials**	**Primary care trial**	**Cardiology trial**
Inclusion criteria	Patients receiving care from MedSeq Project physician participants	Age 40-65 years	Age 18-90 years
		Generally healthy, in the judgment of the patient’s participating physician	Diagnosis of hypertrophic cardiomyopathy (HCM) or dilated cardiomyopathy (DCM)
		No indication for a genetic test	Prior or concurrent targeted genetic testing for HCM or DCM
Exclusion criteria	Clinically significant anxiety (Hospital Anxiety and Depression Scale [HADS] anxiety subscale >14) or depression (HADS depression subscale >16) at baseline assessment		
		Presence of cardiovascular disease or diabetes	
	Reported current pregnancy or intention for future conception in the next year of participant or spouse/partner		

### Study arms

For comparison with the WGS arms, the control arms approximate the standard of care for the two clinical scenarios under study. In routine primary care, the standard of care for identifying the risk of heritable conditions is a general family history assessment. In our cardiomyopathy practice, the standard of care is a family history assessment plus consideration of targeted genetic testing for cardiomyopathy. At the baseline visit of the MedSeq Project, patient participants complete a customized version of the US Surgeon General’s “My Family Health Portrait” web tool [31] to document the diseases diagnosed among their family members. This web tool generates a family history summary based on the patient-entered data. The tool’s designers developed a workflow unique to our study that securely transmits each patient participant’s family history summary to the study staff, who forwards it to the patient’s participating physician. Physician participants also receive six clinical decision support modules to accompany the family history summary for each patient, to assist them in interpreting and managing their patients’ heritable risk of breast and colon cancer, coronary artery disease, type 2 diabetes, glaucoma, and osteoporosis. Patient participants in the control arms undergo only the family history review with their physicians, whereas patient participants randomly assigned to the WGS arms review both their family history summaries and WGS results with their physicians.

### Physician education and support

Many physicians feel ill prepared to address genomic medicine in their clinical practices [32-35], but the current size of the genetics workforce makes it impractical to have a genetic counselor or medical geneticist involved in every instance of genomic medicine as more and more patients are sequenced [36-38]. Moreover, as genomic medicine finds utility in an increasing number of clinical contexts, the most appropriate place for the integration of genomic information may be the existing physician-patient relationship. That is, preventive medicine for healthy adults appropriately belongs in primary care, and the management of diseases such as cardiomyopathy belongs in cardiology. To address the low self-efficacy in genomic medicine that many physicians report, the MedSeq Project provides participating PCPs and cardiologists with an orientation to the study protocol and genome report at the beginning of the study. The educational curriculum consists of two 1-hour in-person group classes and 4 hours of self-paced online modules. The curriculum uses case-based examples to cover general genetics concepts such as inheritance patterns, an overview of Mendelian conditions, genome-wide association studies and risk of common complex diseases, and pharmacogenomics. Participants may receive continuing medical education credits for participation. The MedSeq Project also offers individualized support to physician participants during the course of the study. The MedSeq Project Genome Resource Center (GRC) links physician participants via telephone or e-mail to genetics counselors and medical geneticists affiliated with the study, in a manner similar to hotlines provided by state laboratories to support pediatricians with questions about newborn screening results. Physician participants have the option to contact the GRC with specific questions about the genome reports or family history summaries of their participating patients but are not required to do so. The GRC staff records the reasons that physician participants contact the GRC and any recommendations or actions the GRC staff takes as a result, using a web-based logbook with RedCap™ software [39].

### Whole genome sequencing and analysis

The MedSeq Project models the delivery of WGS in a traditional clinical setting. That is, as in other tests ordered in clinical care, a molecular genetics laboratory analyzes and interprets the WGS data and delivers a report to the physician, who then discusses the results with the patient and develops a management strategy. For the MedSeq Project, genome sequencing is performed at the Illumina Clinical Services Laboratory (San Diego, CA, USA) on the HiSeq 2000 platform [40]. Genomes are sequenced to at least 30× mean coverage, with a minimum of 95% of bases sequenced to at least 8× coverage. Two blood samples are collected from each patient participant; one is sent directly to Illumina for sequencing, and the other is retained by the Laboratory for Molecular Medicine (LMM) for individual variant confirmation, as needed. Once sequencing is complete, Illumina transfers the sequence alignment and variant calling data to the LMM for further analysis via an encrypted hard drive. All analyses are performed in laboratories approved by Clinical Laboratory Improvement Amendments (CLIA).

The data files from each individual genome contain approximately 3 million variants. Geneticists at the LMM prioritize the variants from each genome for further analysis by using two different bioinformatics filtering strategies. The Genome Report filter identifies (a) variants classified as disease-causing mutations in the Human Gene Mutation Database (HGMD) (professional version) [41]; (b) nonsense, frameshift, and ±1,2 canonical splice-site variants with a minor allele frequency of less than 5% in European American or African American chromosomes from the National Heart, Lung and Blood Institute Exome Sequencing Project [42]; (c) pharmacogenomic variants associated with commonly used medications; and (d) a subset of blood group antigens predicted by the genome sequence and confirmed via traditional serological testing of a separate patient sample. In addition, patients in the cardiomyopathy arm have another filter applied, which identifies all variants in 102 preselected monogenic cardiovascular disease genes. This includes variants that would not be identified in filters (a) and (b) above. In the Cardiac Risk Supplement, genotypes at a number of predefined loci are returned for use in algorithms to define risk status for common complex cardiovascular phenotypes.

The LMM staff reviews the scientific evidence for disease causality for each variant that results from the filtering strategies above, with the exception of the predefined set of pharmacogenomics variants and common non-Mendelian risk variants for common complex cardiovascular phenotypes. For candidate monogenic disease variants, analysis components include genetic and functional evidence from primary scientific literature, allele frequency, conservation of affected amino acid or nucleotide (or both), affected protein domain, pathogenicity predictions, and splicing predictions. Finally, each variant is classified according to LMM criteria for pathogenicity [43]. Variants meeting criteria for report inclusion as discussed in the subsequent section are confirmed via a traditional Sanger sequencing method using extracted DNA from the blood sample retained by the LMM.

### The genome report

In the MedSeq Project, the LMM delivers WGS results to physician participants as a Genome Report and a more exploratory Cardiac Risk Supplement, both described below (Table 2) and exemplified in Additional files 1 and 2. Because the target audience of these reports consists of busy clinicians with variable expertise in genetics, the results of the Genome Report are completely summarized on the first page of the report, and subsequent pages contain greater detail about each reported variant. The Genome Report includes results of more general significance, including known pathogenic or likely pathogenic Mendelian variants, carrier status for Mendelian diseases, and known pharmacogenomics associations for five commonly used medications. Owing to the low prior probability of Mendelian disease among the patient participants, variants of uncertain significance (VUSs) in known Mendelian genes are included on the Genome Report if available evidence favors pathogenicity and if clinical follow-up may resolve the variant’s significance or if the variant is in a gene associated with the etiology of the patient participant's disease (HCM or DCM). Because of the frequency of clinical decision-making around cardiovascular disease prevention in primary care, we are using the Cardiac Risk Supplement and cardiovascular disease to explore a wider range of uncertainty in clinical utility than is present for established Mendelian genes. Thus, to supplement the Genome Report, the Cardiac Risk Supplement contains common alleles associated with eight cardiometabolic traits from genome-wide association studies (GWAS), such as coronary artery disease, type 2 diabetes, and atrial fibrillation. We are also piloting the use of a polygenic prediction model for lipid levels.

**Table 2:** Categories of whole genome sequencing results that may be included in the genome report and cardiac risk supplement in the MedSeq project

**Genome report**	**Cardiac risk supplement**
Known pathogenic or likely pathogenic Mendelian variants, including some high-grade variants of uncertain significance resolvable by clinical evaluation	Predicted lipid profile (fasting low-density lipoprotein and high-density lipoprotein cholesterol and triglycerides) derived from polygenic model
Carrier status for Mendelian diseases	Aggregate genetic risk associated with eight cardiometabolic traits from genome-wide association studies
Pharmacogenomic associations for five commonly used medications:	Aortic aneurysm
Warfarin	Atrial fibrillation
Clopidogrel	Coronary artery disease
Digoxin	Hypertension
Metformin	Obesity
Simvastatin	Platelet aggregation
Genetic prediction of blood type with partial serological confirmation	QT prolongation
	Type 2 diabetes

For the cardiometabolic phenotypes known to be relevant both to primary care and cardiology practice, we use GWAS results to aggregate risk information across independent loci to convey a single summary of one’s genetic risk for a given trait. For each trait, the Cardiac Risk Supplement provides multiplicative polygenic risk scores (MPRSs) derived from 161 published risk alleles with small or moderate effects sizes (median odds ratio 1.14). We calculate the MPRS as the product of the odds ratios per risk allele at each of several loci, each raised to its count (*that is,* 0, 1, or 2). The Cardiac Risk Supplement communicates this risk as a polygenic relative risk and its decile after normalizing the MPRS by the population median from the 379 Europeans in the 1000 Genomes Project [44].

Neither the Genome Report nor its Cardiac Risk Supplement includes specific clinical recommendations on follow-up testing or referrals. This is consistent with typical clinical laboratory reports and important for the goals of the MedSeq Project, as we hope to understand how clinicians contextualize and use such information in their practices. The format of the report may evolve over the course of the study, depending on feedback from the physician participants, advances in genomic discovery, and the emergence of more robust population data on which to base clinical risk prediction. Reports are generated by using the GeneInsight Laboratory software system and transmitted to the GeneInsight Clinic system, which is integrated with our institution’s electronic health record (EHR) [45].

### Study schema

Figure 1 shows the study flow and data collection points of the MedSeq Project. At the baseline visit with study staff, patient participants complete the baseline surveys and online family history assessment, undergo a blood draw, and are block-randomized to either the WGS or control arm. In the primary care trial, randomization is sex-matched. In the cardiology trial, randomization is stratified on the basis of previous targeted genetic testing results, such that a known pathogenic variant will have already been identified in approximately half of each randomized arm. No molecular diagnosis has been made for the remaining half, despite their having also undergone targeted cardiomyopathy genetic testing as a part of clinical care.

Once a patient’s family history summary and Genome Report are prepared, generally after a couple of months, they are sent to the physician participant, who then has the option of contacting the GRC for assistance in interpreting the results. The study staff schedules a one-on-one disclosure visit between the patient and physician participants. At this disclosure visit, the patient learns whether he or she was randomly assigned to receive WGS. The patient discusses with his or her physician the findings in the family history summary and, if randomly assigned to receive WGS, the Genome Report. The physician and patient make management decisions about these findings as they would in usual clinical care, which may include pursuing additional follow-up testing or referrals to subspecialists. The physician participant documents the family history and WGS information and the related clinical decision-making in the EHR as he or she feels appropriate. The disclosure visits are audio-recorded for qualitative analysis of physician-patient communication.

Within 1 week after this visit, physician participants are prompted by e-mail to complete a brief RedCap™ -based checklist of the clinical actions they made as a result of the family history summary and Genome Report for a given patient. Patient participants are surveyed immediately after the disclosure visit and 6 weeks and 6 months after disclosure. A subset of 40 patient participants (approximately 10 in each arm of the two trials) undergoes semi-structured interviews at enrollment and then 6 months after their disclosure visits. Each physician participant undergoes an interview at the beginning of the study before patient enrollment and then again after conducting several disclosure visits.

### Study outcomes

The MedSeq Project is leveraging multidisciplinary expertise and a diverse set of tools to collect and analyze data on patients and physicians about a variety of outcomes. Study outcomes focus on six broad domains identified as research priority areas across studies funded by the National Human Genome Research Institute (NHGRI): (a) attitudes and preferences, (b) understanding, (c) psychological impact, (d) behavioral impact, (e) health-care utilization, and (f) decisional satisfaction. When available, we use validated instruments and harmonize the measurements across other NHGRI-funded studies of the application of genome sequencing to clinical care. Longitudinal measurements of many of these domains allow us to examine changes over the study period. Table 3 summarizes the specific outcomes being assessed for each of these domains and the data sources used. Data collection, including questionnaires, interviews, and audio-recorded interactions with patients, is performed at multiple time points. Paper and web-based surveys of patient and physician participants provide self-reported quantitative data about beliefs, attitudes, expectations, psychological states, and intentions. Semi-structured interviews with patients and physician participants allow both groups to describe in their own words their motivations for participation, their attitudes and expectations, and how they responded to results. Audio recordings of the informed consent process and the results disclosure encounter provide *in vivo* data about the kinds of questions that arise and issues that are discussed around WGS. Physician checklists after each disclosure visit, logbooks of physician interactions with the GRC, and reviews of patient participant EHR data provide additional information about service utilization, information seeking, and health status. We believe this mixed-methods approach allows us to achieve significant depth in chronicling the impact of WGS information in the present trial while simultaneously developing processes that will inform the design of future trials. The MedSeq Project Publications Subcommittee has established guidelines for publications and presentations and will review all publications and presentations for consistency with these guidelines and will make recommendations to the Executive Committee regarding the dissemination of study results to relevant stakeholder groups. We will report results according to the CONSORT Statement and its extension for non-pharmacologic treatment interventions and will include a description of how missing data were handled [46,47].

**Table 3:** Patient- and physician-oriented outcomes by domain and data source in the MedSeq project

	**Data source**							
	**Patient surveys**	**Patient interviews**	**Physician surveys**	**Physician interviews**	**Physician checklist**	**Audio recordings**	**EHR review**	**GRC logbook**
**Attitudes and preferences**								
Attitudes about project [48]	✓	✓		✓				
Attitudes about sequencing [13]	✓	✓		✓				
Perceived utility	✓	✓	✓	✓	✓			
Preferences for information	✓	✓						
**Understanding**								
Understanding of consent [49,50]	✓	✓	✓	✓		✓		
Understanding of results	✓	✓				✓		
Genetic self-efficacy [51]	✓		✓					
Genetic literacy [52]	✓	✓	✓	✓				
Health and risk perceptions [53]	✓							
**Psychological impact**								
General anxiety and depression [30]	✓							
Results-specific affect [54]	✓							
Intolerance of uncertainty [55]	✓							
**Behavioral impact**								
Health behaviors and intentions [56]	✓	✓			✓			
Insurance coverage [57]	✓	✓						
Medication and supplement use	✓	✓			✓		✓	
Information seeking and sharing [58]	✓	✓		✓	✓			✓
**Health-care utilization**								
Willingness to pay	✓		✓					
Shared decision-making [59]	✓	✓		✓		✓		
Follow-up testing and screening [60]	✓	✓		✓	✓	✓	✓	✓
**Decisional satisfaction**								
Satisfaction [61]	✓	✓		✓				
Decisional regret [62]	✓	✓		✓				
Expectations	✓	✓		✓				

References indicate published scales used in the design of the data collection instruments for the MedSeq Project. EHR, electronic health record; GRC, Genome Resource Center.

## Discussion

The MedSeq Project consists of two randomized controlled trials designed to model two archetypal situations in genomic medicine and study the impact of integrating genome sequencing into patient care. In the cardiology trial, we want to know how WGS, with a particular focus on cardiac risk information, might alter the ongoing clinical management of cardiomyopathy patients. The cardiology trial also allows us to study how physicians and patients manage incidental genomic findings not directly related to the original reason for genetic testing [27]. In the primary care trial, we want to know how WGS might shape the attitudes, behaviors, and health care of generally healthy individuals. To answer these questions, we have developed a scalable pipeline to analyze and interpret WGS results and deliver a clinically useful genome report to physicians caring for these patients. We provide non-geneticist physician participants with educational and consultative resources that we believe comprise a workable model to support their use of genomic medicine in an era when the demand for genetics specialists exceeds supply.

The design of the MedSeq Project reflects many of the competing priorities inherent in a study of integrating WGS into clinical practice. We know that the field of genomics itself is rapidly evolving over the course of the study, let alone by the time the study’s results are ready for dissemination. This evolution is occurring on many fronts: improvements in sequencing quality, decreasing costs and time necessary to perform sequencing, increasing clinical experience with WGS at many academic medical centers, and continual publication of newly discovered genes and variants linked to human disease. Meanwhile, we are limited by the present technology, costs, and knowledge at the time of study design. We understand that the study of such a moving target risks the collection of results that could rapidly become obsolete, and thus we have modeled processes for the integration of WGS into clinical medicine both now and in the near future. For example, while we are generating genome reports based on the current state of the science and delivering them to the physician-patient encounter, we plan to allow the format and content of these reports to evolve with the field, incorporating new discoveries and refining our interpretations of pathogenicity and utility. We hope to create a paradigm for the clinical integration of WGS which will remain relevant even as genomic medicine advances in technology and content.

We also are attempting to integrate the clinical uncertainty that necessarily accompanies results from WGS, particularly in healthy adults, and at the same time present a meaningful genome report to physicians and their patients. When clinical significance is uncertain, the report will have to acknowledge and communicate this uncertainty effectively, allowing the physician to contextualize the information for the individual patient. This tension necessitates finding the right balance between embracing the ambiguity of WGS results and limiting the scope of what is reported to physician and patient participants. The importance of cardiovascular disease in primary and subspecialty care has prompted us to explore, through the Cardiac Risk Supplement, a greater degree of genomic uncertainty in that disease area as a demonstration of integrating GWAS-type results into clinical care.

In addition, we seek to balance the tensions of providing enough support to participating physicians while minimizing the burden to the limited genetics referral resources available at most medical centers. We have designed the MedSeq Project with the premise that all physicians will soon have to manage at least some genomic information in their practices. Thus, we have intentionally not provided a level of counseling or consultant support that would make our model logistically or economically unsustainable as WGS is increasingly introduced to the clinical context. Still, we acknowledge the need to provide at least some educational and consultation support, both to ensure the quality of care in this novel situation and to monitor for patient safety. We believe that the product of these tensions, the MedSeq Project educational curriculum and Genome Resource Center, represents a workable model for physician support in genomic medicine research, with potential for scaling to routine clinical use.

Our study has some limitations inherent in a trial of this nature. Because we are studying the way that new genomic information is used in clinical care, we cannot blind our patient and physician participants to their randomization status. Unblinded randomized controlled trials may be biased by changes in patient and physician behaviors and outcomes unrelated to the effect of the intervention studied. In our case, however, these changes in behaviors and outcomes are precisely what we are interested in studying. Similarly, our researchers are not blinded to patient randomization. For this reason, our use of objective survey instruments and EHR data is particularly helpful in comparing those who received WGS with those who did not. Finally, each physician participant in the study will have patients in each of the two study arms. Our results will therefore have to be interpreted with the risk that patient outcomes may be correlated with physician behavior in mind. As appropriate, we will perform certain analyses with clustering by physician to account for these possible within-physician effects.

The MedSeq Project is the first randomized controlled trial of WGS in clinical care. We are using a multidisciplinary team of laboratory and clinical geneticists, bioinformaticians, biostatisticians, clinicians, and social scientists to study the many ways that WGS will impact patient care in two archetypal clinical scenarios: disease-specific genomic medicine and general genomic medicine. We expect to produce quantitative and qualitative data that inform the ongoing real-time clinical integration of WGS and also generate novel hypotheses to inform the design of larger studies moving forward. Our considerations of how best to present complex but clinically relevant information derived from WGS to physicians and their patients and measure its impact on clinical care should remain instructive for future research in genomic medicine.

## Trial status

Physician and patient participants were being enrolled as of March 2014.

## Abbreviations

DCM: dilated cardiomyopathy; EHR: electronic health record; GRC: Genome Resource Center; GWAS: genome-wide association study; HADS: hospital anxiety and depression scale; HCM: hypertrophic cardiomyopathy; LMM: laboratory for molecular medicine; MPRS: multiplicative polygenic risk score; NHGRI: National Human Genome Research Institute; PCP: primary care physician; VUS: variant of uncertain significance; WGS: whole genome sequencing.

## Competing interests

CAM receives a modest royalty income from genetic testing for cardiomyopathies. HLR and HMM are employed by Partners Healthcare, which offers fee-for-service molecular diagnostic services (Laboratory for Molecular Medicine) and software licensing (GeneInsight). HLR also serves on compensated advisory boards for Complete Genomics (Mountain View, CA, USA), Knome (Cambridge, MA, USA), and Ingenuity (Redwood City, CA, USA). All other authors declare that they have no competing interests.

## Authors’ contributions

JLV made a substantial contribution to study design and wrote the manuscript. DML, KDC, JK, ISK, LZF, JB-B, JSR, LSL, CYH, PAU, CAM, CES, MFM, ALM, HLR, and RCG each made substantial contributions to the study design and revised the manuscript. HLR, HMM, and SWK made substantial contributions to the genome analysis and interpretation process and genomic report design and edited the manuscript. All authors read and approved the final manuscript.

## Supplementary Material

Additional file 1: Figure S1 Sample Genome Report. Click here for file

Additional file 2: Figure S2 Sample Cardiac Risk Supplement. Click here for file
