# Supplementary material for: The MedSeq Project: a randomized trial of integrating whole genome sequencing into clinical medicine
Source: Trials. 2014 Mar 20;15:85. doi: 10.1186/1745-6215-15-85 (PMC4113228; doi:10.1186/1745-6215-15-85)
Supplement: Additional file 1: Figure S1 — Sample Genome Report. [file 1745-6215-15-85-S1.pdf]

LABORATORY FOR MOLECULAR MEDICINE
65 Landsdowne Street, Cambridge, MA 02139
Phone: (617)768-8500 / Fax: (617)768-8513
http://pcpgm.partners.org/lmm

PARTNERS HEALTHCARE

CENTER FOR PERSONALIZED GENETIC MEDICINE

A teaching affiliate of: HARVARD MEDICAL SCHOOL

Name: DOE, JONATHAN

Accession ID: PMXX-12345

DOB: 12/34/5678
MRN:123456789
Family #: F12345
Sex: Male
Specimen: Blood, Peripheral
Referring physician: MedSeq
Race: Caucasian
Received: 12/34/5678
Referring facility: MedSeq

GENERAL GENOME REPORT

RESULT SUMMARY

Sequencing of this individual's genome was performed and covered 95.7% of all positions at 8X coverage or higher, resulting in over 5.2 million variants compared to reference genome. These data were analyzed to identify previously reported variants of potential clinical relevance as well as novel variants that could reasonably be assumed to cause disease (see methodology below). All results are summarized on page 1 with further details on subsequent pages.

MONOGENIC DISEASE RISK: 1 VARIANT IDENTIFIED

This test identified 1 genetic variant that may be responsible for existing disease or the development of disease in this individual's lifetime.

Disease (Inheritance)	Phenotype	Gene (Variant)	Classification
X-linked recessive chondrodysplasia punctata (X-linked)	Abnormal bone and cartilage development	ARSE (c.410G>C p.Gly137Ala)	Uncertain significance: Favor pathogenic

CARRIER RISK: 2 VARIANTS IDENTIFIED

This test identified carrier status for 2 autosomal recessive disorders.

Disease (Inheritance)	Phenotype	Gene (Variant)	Classification	Carrier Phenotype*
Cystic fibrosis (Autosomal recessive)	Chronic lung and digestive disease	CFTR (c.3846G>A p.Trp1282X)	Pathogenic	None Reported
Glycogen storage disease 7 (Autosomal recessive)	Severe exercise intolerance	PFKM (c.237+1G>A)	Pathogenic	None Reported

As a carrier for recessive genetic variants, this individual is at higher risk for having a child with one or more of these highly penetrant disorders. To determine the risk for this individual's future children to be affected, the partner of this individual would also need to be tested for these variants. Other biologically related family members may also be carriers of these variants. *Carriers for some recessive disorders may be at risk for certain phenotypes. Please see variant descriptions for more information.

PHARMACOGENOMIC ASSOCIATIONS

This test identified the following pharmacogenomic associations. Additional pharmacogenomic results may be requested, but will require additional molecular confirmation prior to disclosure.

Drug	Risk and Dosing Information
Warfarin	Increased dose requirement
Clopidogrel	Typical response to clopidogrel
Digoxin	Intermediate metabolism and serum concentration of digoxin
Metformin	Decreased glycemic response to metformin
Simvastatin	Typical risk of simvastatin-related myopathy

BLOOD GROUPS

This test identified the ABO Rh Blood Type as AB Negative. Based on their results, this person is a very desirable universally compatible platelet donor. Additional RBC and platelet antigen information is available at the end of the report.

It should be noted that the disease risk section of this report is limited only to variants with strong evidence for causing highly penetrant disease, or contributing to highly penetrant disease in a recessive manner. Not all variants identified have been analyzed, and not all regions of the genome have been adequately sequenced. These results should be interpreted in the context of the patient's medical evaluation, family history, and racial/ethnic background. Please note that variant classification and/or interpretation may change over time if more information becomes available. For questions about this report, please contact the Genome Resource Center at GRC@partners.org.

DETAILED VARIANT INFORMATION

MONOGENIC DISEASE RISK

Disease (Inheritance)	Gene (Transcript)	Variant (Classification)	Variant Frequency	Disease Prevalence	References
X-linked recessive chondrodysplasia punctata (X-linked)	ARSE (NM_000047.2)	c.410G>C p.Gly137Ala hemizygous (Uncertain significance: Favor pathogenic)	1/6728 (0.01%) European American	1:500,000	Sheffield 1998, Nino 2008, Franco 1995, Matos-Miranda 2013

VARIANT INTERPRETATION: The Gly137Ala variant in ARSE has been previously identified in 2 males with chondrodysplasia punctata; however, this variant was also identified in one unaffected male family member (Sheffield 1998, Nino 2008). Variants in a paralogous gene (ARSB) at the same position have also been identified in an individual with Maroteux-Lamy syndrome, which also features skeletal abnormalities (Franco 1995). Functional studies indicate that the Gly137Ala variant leads to reduced ARSE activity (Matos-Miranda 2013). In summary, although some data support a disease-causing role, there is currently insufficient evidence for pathogenicity leading to a current classification of uncertain significance.

DISEASE INFORMATION: X-linked chondrodysplasia punctata 1 (CDPX1), a congenital disorder of bone and cartilage development, is caused by a deficiency of the Golgi enzyme arylsulfatase E (ARSE). It is characterized by chondrodysplasia punctata (stippled epiphyses), brachytelephalangy (shortening of the distal phalanges), and nasomaxillary hypoplasia. Although most affected males have minimal morbidity and skeletal findings that improve by adulthood, some have significant medical problems including respiratory compromise, cervical spine stenosis and instability, mixed conductive and sensorineural hearing loss, and abnormal cognitive development. From GeneReviews abstract: http://www.ncbi.nlm.nih.gov/books/NBK1544/

FAMILIAL RISK: X-Linked chondrodysplasia punctata is inherited in an X-linked recessive manner, with primarily males being affected. Each child is at a 50% (or 1 in 2) chance of inheriting the variant from a carrier female, while all daughters will inherit the variant from an affected male.

CARRIER RISK

Disease (Inheritance)	Gene (Transcript)	Variant (Classification)	Variant Frequency	Disease Prevalence (Carrier Freq.)	References	Carrier Phenotype
Cystic fibosis (Autosomal recessive)	CFTR (NM_000492.3)	c.3846G>A p.Trp1282X heterozygous (Pathogenic)	6/8600 (0.07%) European American	1/3200 European American (1/25)	Hamosh 1991, Kerem 1990, Shoshani 1992, Vidaud 1990	None Reported

VARIANT INTERPRETATION: The Trp1282X variant in CFTR has been identified in numerous patients with cystic fibrosis (Viduad 1990, Kerem 1990, Hamosh 1991, Shoshani 1992). This variant is present on the American Board of Medical Genetics CFTR mutation panel (http://www.acmg.net/Pages/ACMG_Activities/stds-2002/cf.htm). This nonsense variant leads to a premature termination codon at position 1282, which is predicted to lead to a truncated or absent protein. In summary, this variant meets our criteria for pathogenicity.

DISEASE INFORMATION: Cystic fibrosis effects the epithelia of the respiratory tract, exocrine pancreas, intestine, male genital tract, hepatobiliary system, and exocrine sweat glands, resulting in a complex multisystem disease. Pulmonary disease is the major cause of morbidity and mortality in CF. Affected individuals have lower airway inflammation and chronic endobronchial infection, progressing to end-stage lung disease characterized by extensive airway damage (bronchiectasis, cysts, and abscesses) and fibrosis of lung parenchyma. Meconium ileus occurs at birth in 15%-20% of newborns with CF. Pancreatic insufficiency with malabsorption occurs in the great majority of individuals with CF. More than 95% of males with CF are infertile as a result of azoospermia caused by absent, atrophic, or fibrotic Wolffian duct structures. Adapted from GeneReviews abstract: http://www.ncbi.nlm.nih.gov/books/NBK1250/

FAMILIAL RISK: Cystic fibrosis is inherited in an autosomal recessive manner. A carrier of cystic fibrosis has a 50% chance of passing on the CFTR variant to any children. The risk of this patient’s child having cystic fibrosis is dependent on the CFTR carrier status of the patient’s partner. This patient likely inherited the CFTR variant from one of his parents. Other biologically related family members may also be carriers of this variant.

Disease (Inheritance)	Gene (Transcript)	Variant (Classification)	Variant Frequency	Disease Prevalence (Carrier Freq.)	References	Carrier Phenotype
Glycogen storage disease 7 (Autosomal recessive)	PFKM (NM_000289.5)	c.237+1G>A heterozygous Pathogenic	Not previously reported	Unknown (Unknown)	Raben 1993	None Reported

VARIANT INTERPRETATION: The 237+1G>A variant in PFKM has been previously identified in one homozygous patient with glycogen storage disease 7 and was found to segregate with disease in an affected homozygous relative (Raben 1993). This variant is located in the 5' splice region and computational tools do suggest an impact to splicing. In summary, this variant meets our criteria for pathogenicity.

DISEASE INFORMATION: Glycogen storage disease 7 is caused by a deficiency of muscle phosphofructokinase activity. Symptoms usually appear in adulthood and are characterized by exercise intolerance with muscle cramps that can be accompanied by attacks of myoglobinuria. Some patients also experience compensated hemolytic anemia and early onset myogenic hyperuricemia. In addition to the accumulation of normal glycogen in muscle, an abnormal glycogen, resembling amylopectin, can be found in some muscle fibers. Adapted from Online Metabolic and Molecular Basis of Inherited Disease abstract:
http://www.ommbid.com//OMMBID/the_online_metabolic_and_molecular_bases_of_inherited_disease/b/abstract/part7/ch71

FAMILIAL RISK: Glycogen storage disease 7 is inherited in an autosomal recessive manner. A carrier of glycogen storage disease 7 has a 50% chance of passing on the PFKM variant to any children. The risk of this patient's child having Glycogen storage disease 7 is dependent on the PFKM carrier status of the patient's partner. This patient likely inherited the PFKM variant from one of his parents. Other biologically related family members may also be carriers of this variant.

PHARMACOGENOMIC ASSOCIATIONS AND BLOOD GROUPS

PHARMACOGENOMIC ASSOCIATIONS

Drug (Indication)	Summary	Variants Evaluated and Genotypes Identified	Interpretation	References
Warfarin (Anti-coagulation)	**Standard dose requirement**	CYP2C9 rs1057910 rs1799853 Genotype: c.[430C;1075A]; c.[430C>T;1075A] *1/*2<br><br>VKORC1 rs9923231 Genotype: AA	Patients with the CYP2C9*1/*2 genotype may require a lower dose of warfarin as compared to patients with the CYP2C9*1/*1 genotype. Patients with the VKORC1 AA genotype may require a lower dose of warfarin as compared to patients with the VKORC1 GG or GA genotypes. However, patients with the combination of the CYP2C9*1/*2 genotype and VKORC1 AA genotype are predicted to require standard doses of warfarin compared to other patients. Refer to warfarindosing.org for dosing based on genotype and other clinical factors.	Takeuchi 2009, Cooper 2008, International Warfarin Pharmaccogenetics Consortium 2009, Margaglione 2000, Pautas 2010, Scott 2010, Verstuyft 2001

GENERAL GENOME REPORT(CONTINUED)

VKORC1/CYPC2C9 GENOTYPE COMBINATION FREQUENCIES

Dosing Group	VKORC1 rs9923231	CYPC2C9 Genotypes	Approximate Frequency
Lower	AA	*1/*3, *2/*2, *2/*3, *3/*3	6%
	GA	*2/*3, *3/*3	3%
Standard	AA	*1/*1, *1/*2	37%
	GA	*1/*2, *1/*3, *2/*2	14%
	GG	*1/*3, *2/*2, *2/*3	‹1%
Higher	GA	*1/*1	28%
	GG	*1/*1, *1/*2	13%

Clopidogrel (Anti-coagulation)	**Typical response to clopidogrel**	CYP2C19<br>rs12248560<br>rs4244285<br>rs4986893<br>Genotype: *1/*1<br>c.[-806C(;)681G(;)636G];<br>c.[-806C(;)681G(;)636G]	Patients with the CYP2C19 *1/*1 genotype may have extensive (typical) metabolism of clopidogrel as well as well as typical response to clopidogrel as compared to ultrarapid or poor clopidogrel metabolizers. Additional information and dosing recommendations for this result can be found at: http://www.pharmgkb.org/drug/PA449053.	Tiroch 2010, Sim 2006, Sibbing 2010

CYP2C19 GENOTYPE FREQUENCIES

Metabolism	Genotypes	Frequency
Ultrarapid	*1/*17, *17/*17	5-30%
Extensive	*1/*1	35-50%
Intermediate	*1/*2, *1/*3	18-35%
Poor	*2/*2, *2/*3, *3/*3	2-15%

Digoxin (Dysrhythmias, heart failure)	**Intermediate metabolism and serum concentration of digoxin**	ABCB1<br>rs1045642<br>Genotype: CT<br><br>Genotype frequencies:<br>CC:50% **CT:22%**<br>TT:28%	Patients with the CT genotype who take oral digoxin may have intermediate metabolism and serum concentrations of digoxin as compared to patients with the CC and TT genotypes.	Aarnoudse 2008, Kurata 2002, Hoffmeyer 2000
Metformin (Type 2 diabetes mellitus)	**Typical glycemic response to metformin**	C11orf65<br>rs11212617<br>Genotype: TT<br><br>Genotype frequencies:<br>**TT:37%** TG:48%<br>GG:15%	Patients with the TT genotype who have Type 2 Diabetes Mellitus and are treated with metformin may have a decreased glycemic response as compared to patients with the GG genotype. An association with increased or decreased glycemic response to metformin was not seen in people diagnosed with impaired glucose tolerance in the absence of Type 2 Diabetes Mellitus.	Florez 2012, GoDARTS and UKPDS Diabetes Pharmacogenetics Study Group 2011

GENERAL GENOME REPORT(CONTINUED)

Drug (Indication)	Summary	Variants Evaluated and Genotypes Identified	Interpretation	References
Simvastatin (Hyperlipidemia)	**Typical risk of simvastatin-related myopathy**	SLCO1B1<br>rs4149056<br>Genotype: TT<br><br>Genotype frequencies:<br>**TT:68%** TC:30% CC:2%	Patients with the TT genotype may have a lower risk of simvastatin-related myopathy as compared to patients with the CT or CC genotype.	Pasanen 2006, SEARCH Collaborative Group 2008, Brunham 2012

RED BLOOD CELL AND PLATELET ANTIGENS

These red blood cell (RBC) and human platelet antigen (HPA) predictions are based on published genotype to phenotype correlations for the alleles present. Some antigens have also been serologically determined using traditional blood typing methods.

SUMMARY

ABO Rh Blood Type: AB Negative.

RBC Antigens

Antigen	Freq	Comments
AB	[4%]	No risk of alloantibody formation in individual. Very desirable platelet donor.
Yk(a−)	[8%]	Risk of alloantibody formation in individual, but anti-Yk(a) is a clinically insignificant antibody.
D−	[15%]	Risk of alloantibody formation in individual. Desirable antigen negative platelet and RBC donor.
Jk(b−)	[26%]	Risk of alloantibody formation in individual. Desirable antigen negative RBC donor.
E−	[29%]	Risk of alloantibody formation in individual. Desirable antigen negative RBC donor.
C−	[32%]	Risk of alloantibody formation in individual. Desirable antigen negative RBC donor.

Platelet Antigens

Antigen	Freq	Comments
HPA-1(b−)	[73%]	Risk of platelet alloantibody formation in individual. Desirable antigen negative platelet donor for the third most common anti-platelet antigen alloantibody cause of platelet refractoriness and a known cause of Neonatal Alloimmune Thrombocytopenia (FNAIT).

DISCUSSION

During pregnancy or transfusion alloantibodies to blood group antigens and platelet antigens can form against foreign RBCs that contain immunogenic blood group and platelet antigens that the recipient is missing. These alloantibodies can cause clinically important complications during future transfusions and pregnancy.

Blood Production Transfusion

This test identified that this individual is most likely to form the following common alloantibodies: anti-Jk(b), anti-C, anti-E, and anti-HPA-1(b). However, this individual does NOT have an increased risk of forming unusual RBC or platelet alloantibodies, since this test also revealed a normal absence of low frequency antigens, normal presence of high frequency antigens, and no antigen gene rearrangements.

Blood Production Donation

This person is a very desirable universally compatible platelet donor since AB Negative individuals, which make up less than 1% of the population, do not have naturally occurring anti-A and anti-B RBC alloantibodies and their RBCs are missing the D antigen so the small fraction of retained RBCs will not induce anti-D alloantibody formation. This person is also a desirable RBC donor missing the RBC antigens D, Jk(b), C, and E, which are common anti-RBC alloantibody targets. Given the population distribution of the C and E antigens, the combination of a donor being C− and E− is very desirable, since many individuals will form both anti-C and anti-E RBC alloantibodies. They are also a desirable platelet donor missing the platelet antigen HPA-1(b), which is the third most common anti-platelet antigen alloantibody cause of platelet refractoriness and a known cause of Fetal and Neonatal Alloimmune Thrombocytopenia (FNAIT). If interested in becoming a platelet and/or RBC donor, this individual is encouraged to contact the BWH donor recruitment supervisor (Malissa Lichtenwalter 617-632-3206, MLichtenwalter@partners.org) and mention that our testing found them to be ABO Rh Blood Type AB Negative; RBC antigen Jk(b−), E−, and C−; and platelet antigen HPA-1(b−).

GENERAL GENOME REPORT(CONTINUED)

RED BLOOD CELL ANTIGENS

D	C	c	E	e	K	k	Fy(a)	Fy(b)	Jk(a)	Jk(b)	M	N	S	s
-	-	+	-	+	-	+	+	+	+	-	+	+	-	+

Lu(a)	Lu(b)	Au(a)	Au(b)	Kp(a)	Kp(b)	Kp(c)	Di(a)	Di(b)	Wr(a)	Wr(b)	Yt(a)	Yt(b)	Sc1	Sc2
[-]	[+]	[+]	[-]	[-]	[+]	[-]	[-]	[+]	[-]	[+]	[+]	[-]	[+]	[-]

Do(a)	Do(b)	Jo(a)	Hy	Co(a)	Co(b)	LW(a)	LW(b)	Cr(a)	Kn(a)	Kn(b)	Sl(a)	Vil	Yk(a)	KCAM
[-]	[+]	[+]	[+]	[+]	[-]	[+]	[-]	[+]	[+]	[-]	[+]	[-]	[-]	[+]

McC(a)	McC(b)	In(a)	In(b)	Ok(a)	MER2	JMHK	JMHL	FORS	A	B	H
[+]	[-]	[-]	[+]	[+]	[+]	[+]	[+]	[-]	+	+	+

PLATELET ANTIGENS

1a	1b	2a	2b	3a	3b	4a	4b	5a	5b	6bw	7bw	8bw	9bw
[+]	[-]	[+]	[-]	[+]	[+]	[+]	[-]	[+]	[+]	[-]	[-]	[-]	[-]

10bw	11bw	12bw	13bw	14bw	15a	15b	16bw	17bw	18bw	19bw	20bw	21bw	22bw
[-]	[-]	[-]	[-]	[-]	[+]	[+]	[-]	[-]	[-]	[-]	[-]	[-]	[-]

23bw	24bw	25bw	26bw	27bw
[-]	[-]	[-]	[-]	[-]

Key: [+] presence of antigen predicted by genotyping; + presence of antigen predicted by genotyping and confirmed by serology; +* presence of antigen detected by serology, genotype prediction not available; [+w] weak presence of antigen predicted by genotyping; +w weak presence of antigen predicted by genotyping and confirmed by serology; +w* weak presence of antigen detected by serology, genotype prediction not available; [−] absence of antigen predicted by genotyping; - absence of antigen predicted by genotyping and confirmed by serology, -* absence of antigen detected by serology, genotype prediction not available; NC indicates no sequencing coverage, Dis indicates discordant. Rare (less than 5% population frequency) presence or absence of antigen is indicated in red.

METHODOLOGY

Genomic sequencing is performed using next generation sequencing on the Illumina HiSeq platform. Genomes are sequenced to at least 30X mean coverage and a minimum of 95% of bases are sequenced to at least 8X coverage. Paired-end 100bp reads are aligned to the NCBI reference sequence (GRCh37) using the Burrows-Wheeler Aligner (BWA), and variant calls are made using the Genomic Analysis Tool Kit (GATK). Variants are subsequently filtered to identify: (1) variants classified as disease causing in public databases; (2) nonsense, frameshift, and +/-1,2 splice-site variants that are novel or have a minor allele frequency <1% in European American or African American chromosomes from the NHLBI Exome Sequencing Project (http://evs.gs.washington.edu/EVS/); and (3) rs11212617 (C11orf65; metformin), rs12248560 (CYP2C19; clopidogrel), rs4244285 (CYP2C19; clopidogrel), rs4986893 (CYP2C19; clopidogrel), rs28399504 (CYP2C19; clopidogrel), rs41291556 (CYP2C19; clopidogrel), rs72552267 (CYP2C19; clopidogrel), rs72558186 (CYP2C19; clopidogrel), rs56337013 (CYP2C19; clopidogrel), rs1057910 (CYP2C9; warfarin), rs1799853 (CYP2C9; warfarin), rs7900194 (CYP2C9; warfarin), rs9332131 (CYP2C9; warfarin), rs28371685 (CYP2C9; warfarin), rs28371686 (CYP2C9; warfarin), rs9923231 (VKORC1; warfarin), rs4149056 (VKORC1; simvastatin), and rs1045642 (ABCB1; digoxin). The evidence for phenotype-causality is then evaluated for each variant resulting from the filtering strategies above and variants are classified according to LMM criteria (http://pcpgm.partners.org/LMM). Only those variants with evidence for causing highly penetrant disease or contributing to disease in a recessive manner are reported. Before reporting, all variants are confirmed via Sanger sequencing or another orthogonal technology. The initial sequencing component of this test was performed by the Illumina Clinical Services Laboratory (San Diego, CA CLIA# 05D1092911) and the alignment, variant calling, data filtering, Sanger confirmation and interpretation were performed by the Laboratory for Molecular Medicine at the Partners Healthcare Center for Personalized Genetic Medicine (Cambridge, MA CLIA#22D1005307). This test has not been cleared or approved U.S. Food and Drug Administration (FDA). The FDA has determined that such clearance or approval is not necessary.

LIMITATIONS

It should be noted that this test does not sequence all bases in a human genome and not all variants have been identified or interpreted. Triplet repeat expansions, translocations and large copy number events are currently not reliably detected by genome sequencing. Furthermore, not all disease-associated genes have been identified and the clinical significance of variation in many genes is not well understood. It is recommended that genomic sequencing data is periodically reinterpreted, especially when new symptoms arise.

REFERENCES

Aarnoudse AJ, Dieleman JP, Visser LE, Arp PP, van der Heiden IP, van Schaik RH, Molokhia M, Hofman A, Uitterlinden AG, Stricker BH. 2008. Common ATP-binding cassette B1 variants are associated with increased digoxin serum concentration. Pharmacogenet. Genomics. 18(4):299-305.

Brunham LR, Lansberg PJ, Zhang L, Miao F, Carter C, Hovingh GK, Visscher H, Jukema JW, Stalenhoef AF, Ross CJ, Carleton BC, Kastelein JJ, Hayden MR. 2012. Differential effect of the rs4149056 variant in SLCO1B1 on myopathy associated with simvastatin and atorvastatin. Pharmacogenomics J. 12(3):233-7.

Cooper GM, Johnson JA, Langaee TY, Feng H, Stanaway IB, Schwarz UI, Ritchie MD, Stein CM, Roden DM, Smith JD, Veenstra DL, Rettie AE, Rieder MJ. 2008. A genome-wide scan for common genetic variants with a large influence on warfarin maintenance dose. Blood. 112(4):1022-7.

Florez JC, Jablonski KA, Taylor A, Mather K, Horton E, White NH, Barrett-Connor E, Knowler WC, Shuldiner AR, Pollin TI, Diabetes Prevention Program Research Group. 2012. The C allele of ATM rs11212617 does not associate with metformin response in the Diabetes Prevention Program. Diabetes Care. 35(9):1864-7.

Franco B, Meroni G, Parenti G, Levilliers J, Bernard L, Gebbia M, Cox L, Maroteaux P, Sheffield L, Rappold GA, Andria G, Petit C, Ballabio A. 1995. A cluster of sulfatase genes on Xp22.3: mutations in chondrodysplasia punctata (CDPX) and implications for warfarin embryopathy. Cell. 81(1):15-25.

GoDARTS and UKPDS Diabetes Pharmacogenetics Study Group, Wellcome Trust Case Control Consortium 2, Zhou K, Bellenguez C, Spencer CC, Bennett AJ, Coleman RL, Tavendale R, Hawley SA, Donnelly LA, Schofield C, Groves CJ, Burch L, Carr F, Strange A, Freeman C, Blackwell JM, Bramon E, Brown MA, Casas JP, Corvin A, Craddock N, Deloukas P, Dronov S, Duncanson A, Edkins S, Gray E, Hunt S, Jankowski J, Langford C, Markus HS, Mathew CG, Plomin R, Rautanen A, Sawcer SJ, Samani NJ, Trembath R, Viswanathan AC, Wood NW, MAGIC investigators, Harries LW, Hattersley AT, Doney AS, Colhoun H, Morris AD, Sutherland C, Hardie DG, Peltonen L, McCarthy MI, Holman RR, Palmer CN, Donnelly P, Pearson ER. 2011. Common variants near ATM are associated with glycemic response to metformin in type 2 diabetes. Nat. Genet. 43(2):117-20.

Hamosh A, Trapnell BC, Zeitlin PL, Montrose-Rafizadeh C, Rosenstein BJ, Crystal RG, Cutting GR. 1991. Severe deficiency of cystic fibrosis transmembrane conductance regulator messenger RNA carrying nonsense mutations R553X and W1316X in respiratory epithelial cells of patients with cystic fibrosis. J. Clin. Invest. 88(6):1880-5.

Hoffmeyer S, Burk O, von Richter O, Arnold HP, Brockmöller J, Johne A, Cascorbi I, Gerloff T, Roots I, Eichelbaum M, Brinkmann U. 2000. Functional polymorphisms of the human multidrug-resistance gene: multiple sequence variations and correlation of one allele with P-glycoprotein expression and activity in vivo. Proc. Natl. Acad. Sci. U.S.A. 97(7):3473-8.

International Warfarin Pharmacogenetics Consortium, Klein TE, Altman RB, Eriksson N, Gage BF, Kimmel SE, Lee MT, Limdi NA, Page D, Roden DM, Wagner MJ, Caldwell MD, Johnson JA. 2009. Estimation of the warfarin dose with clinical and pharmacogenetic data. N. Engl. J. Med. 360(8):753-64.

Kerem BS, Zielenski J, Markiewicz D, Bozon D, Gazit E, Yahav J, Kennedy D, Riordan JR, Collins FS, Rommens JM. 1990. Identification of mutations in regions corresponding to the two putative nucleotide (ATP)-binding folds of the cystic fibrosis gene. Proc. Natl. Acad. Sci. U.S.A. 87(21):8447-51.

Kurata Y, Ieiri I, Kimura M, Morita T, Irie S, Urae A, Ohdo S, Ohtani H, Sawada Y, Higuchi S, Otsubo K. 2002. Role of human MDR1 gene polymorphism in bioavailability and interaction of digoxin, a substrate of P-glycoprotein. Clin. Pharmacol. Ther. 72(2):209-19.

Limdi NA, Wadelius M, Cavallari L, Eriksson N, Crawford DC, Lee MT, Chen CH, Motsinger-Reif A, Sagreiya H, Liu N, Wu AH, Gage BF, Jorgensen A, Pirmohamed M, Shin JG, Suarez-Kurtz G, Kimmel SE, Johnson JA, Klein TE, Wagner MJ, International Warfarin Pharmacogenetics Consortium. 2010. Warfarin pharmacogenetics: a single VKORC1 polymorphism is predictive of dose across 3 racial groups. Blood. 115(18):3827-34.

Margaglione M, Colaizzo D, D'Andrea G, Brancaccio V, Ciampa A, Grandone E, Di Minno G. 2000. Genetic modulation of oral anticoagulation with warfarin. Thromb. Haemost. 84(5):775-8.

Matos-Miranda C, Nimmo G, Williams B, Tysoe C, Owens M, Bale S, Braverman N. 2013. A prospective study of brachytelephalangic chondrodysplasia punctata: identification of arylsulfatase E mutations, functional analysis of novel missense alleles, and determination of potential phenocopies. Genet. Med.

Nino M, Matos-Miranda C, Maeda M, Chen L, Allanson J, Armour C, Greene C, Kamaluddeen M, Rita D, Medne L, Zackai E, Mansour S, Superti-Furga A, Lewanda A, Bober M, Rosenbaum K, Braverman N. 2008. Clinical and molecular analysis of arylsulfatase E in patients with brachytelephalangic chondrodysplasia punctata. Am. J. Med. Genet. A. 146A(8):997-1008.

GENERAL GENOME REPORT(CONTINUED)

Pasanen MK, Neuvonen M, Neuvonen PJ, Niemi M. 2006. SLCO1B1 polymorphism markedly affects the pharmacokinetics of simvastatin acid. Pharmacogenet. Genomics. 16(12):873-9.

Pautas E, Moreau C, Gouin-Thibault I, Golmard JL, Mahé I, Legendre C, Taillandier-Hériche E, Durand-Gasselin B, Houllier AM, Verrier P, Beaune P, Loriot MA, Siguret V. 2010. Genetic factors (VKORC1, CYP2C9, EPHX1, and CYP4F2) are predictor variables for warfarin response in very elderly, frail inpatients. Clin. Pharmacol. Ther. 87(1):57-64.

Raben N, Sherman J, Miller F, Mena H, Plotz P. 1993. A 5' splice junction mutation leading to exon deletion in an Ashkenazic Jewish family with phosphofructokinase deficiency (Tarui disease). J. Biol. Chem. 268(7):4963-7.

Scott SA, Khasawneh R, Peter I, Kornreich R, Desnick RJ. 2010. Combined CYP2C9, VKORC1 and CYP4F2 frequencies among racial and ethnic groups. Pharmacogenomics. 11(6):781-91.

SEARCH Collaborative Group, Link E, Parish S, Armitage J, Bowman L, Heath S, Matsuda F, Gut I, Lathrop M, Collins R. 2008. SLCO1B1 variants and statin-induced myopathy--a genomewide study. N. Engl. J. Med. 359(8):789-99.

Sheffield LJ, Osborn AH, Hutchison WM, Sillence DO, Forrest SM, White SJ, Dahl HH. 1998. Segregation of mutations in arylsulphatase E and correlation with the clinical presentation of chondrodysplasia punctata. J. Med. Genet. 35(12):1004-8.

Shoshani T, Augarten A, Gazit E, Bashan N, Yahav Y, Rivlin Y, Tal A, Seret H, Yaar L, Kerem E. 1992. Association of a nonsense mutation (W1282X), the most common mutation in the Ashkenazi Jewish cystic fibrosis patients in Israel, with presentation of severe disease. Am. J. Hum. Genet. 50(1):222-8.

Sibbing D, Koch W, Gebhard D, Schuster T, Braun S, Stegherr J, Morath T, Schömig A, von Beckerath N, Kastrati A. 2010. Cytochrome 2C19*17 allelic variant, platelet aggregation, bleeding events, and stent thrombosis in clopidogrel-treated patients with coronary stent placement. Circulation. 121(4):512-8.

Sim SC, Risinger C, Dahl ML, Aklillu E, Christensen M, Bertilsson L, Ingelman-Sundberg M. 2006. A common novel CYP2C19 gene variant causes ultrarapid drug metabolism relevant for the drug response to proton pump inhibitors and antidepressants. Clin. Pharmacol. Ther. 79(1):103-13.

Takeuchi F, McGinnis R, Bourgeois S, Barnes C, Eriksson N, Soranzo N, Whittaker P, Ranganath V, Kumanduri V, McLaren W, Holm L, Lindh J, Rane A, Wadelius M, Deloukas P. 2009. A genome-wide association study confirms VKORC1, CYP2C9, and CYP4F2 as principal genetic determinants of warfarin dose. PLoS Genet. 5(3):e1000433.

Tiroch KA, Sibbing D, Koch W, Roosen-Runge T, Mehilli J, Schömig A, Kastrati A. 2010. Protective effect of the CYP2C19 *17 polymorphism with increased activation of clopidogrel on cardiovascular events. Am. Heart J. 160(3):506-12.

Verstuyft C, Morin S, Robert A, Loriot MA, Beaune P, Jaillon P, Becquemont L. 2001. Early acenocoumarol overanticoagulation among cytochrome P450 2C9 poor metabolizers. Pharmacogenetics. 11(8):735-7.

Vidaud M, Fanen P, Martin J, Ghanem N, Nicolas S, Goossens M. 1990. Three point mutations in the CFTR gene in French cystic fibrosis patients: identification by denaturing gradient gel electrophoresis. Hum. Genet. 85(4):446-9.
