# Supplementary material for: The MedSeq Project: a randomized trial of integrating whole genome sequencing into clinical medicine
Source: Trials. 2014 Mar 20;15:85. doi: 10.1186/1745-6215-15-85 (PMC4113228; doi:10.1186/1745-6215-15-85)
Supplement: Additional file 2: Figure S2 — Sample Cardiac Risk Supplement. [file 1745-6215-15-85-S2.pdf]

LABORATORY FOR MOLECULAR MEDICINE
65 Landsdowne Street, Cambridge, MA 02139
Phone: (617)768-8500 / Fax: (617)768-8513
http://pcpgm.partners.org/lmm

Name: DOE, JONATHAN | **Accession ID:** PMXX-67890

DOB: 12/34/5678 | MRN: 123456789 | Family #: F12345
Sex: Male | Specimen: Blood, Peripheral | Referring physician: MedSeq
Race: Caucasian | Received: 12/34/5678 | Referring facility: MedSeq

CARDIAC RISK REPORT

RESULT SUMMARY

This Cardiac Risk Report returns genetic variants that may influence risk or treatment for cardiovascular disease. Analysis covers 102 genes for monogenic cardiac disease including cardiomyopathies, arrhythmias, aortopathies, congenital heart disease as well as vascular and valvular disorders. This report includes pathogenic variants found in these genes (also returned on the General Genome Report). In addition, variants of uncertain significance in these genes are returned in this report. Please note that the following genes had less than 95% coverage at a depth of 8X or higher: CTF1 (94%), HCN2 (89%), and PABPN1 (91%). Fasting lipid levels are predicted based on the presence or absence of multiple genetic variants and, finally, alleles conferring a small to moderate risk for cardiovascular disease are returned from analysis of 161 cardiovascular risk loci. All results are summarized on page 1 with further details provided on subsequent pages.

MONOGENIC CARDIAC DISEASE RISK: 0 VARIANTS IDENTIFIED

This test did NOT identify genetic variants known or expected to cause existing cardiovascular disease or the development of cardiovascular disease in this individual's lifetime.

POSSIBLE MONOGENIC CARDIAC DISEASE RISK: 1 VARIANT IDENTIFIED

This test identified 1 genetic variant of uncertain significance that could be responsible for existing cardiovascular disease or the development of cardiovascular disease in this individual's lifetime.

Disease (Inheritance)	Phenotype	Gene (Variant)	Classification
Brugada syndrome (Autosomal dominant)	Cardiac conduction abnormalities	CACNA1C (c.1468G>A p.Gly490Arg)	Uncertain significance

POLYGENIC PREDICTED FASTING LIPID PROFILE

The following lipid profile is predicted by known genetic factors, age, and gender and is not reflective of environmental, medication or other factors. These values are based on large epidemiologic studies and are not intended to substitute for measured values.

- LDL 116 mg/dL
- HDL 47 mg/dL
- Triglycerides 140 mg/dL

ALLELES CONFERRING SMALL-MODERATE RISK MODIFICATION FOR EIGHT CARDIOVASCULAR PHENOTYPES

	Contextual Data		Patient Results			
Phenotype	Population Prevalence of Phenotype for Age 54	Proportion of Variation in Phenotype Liability Explained by Common Genetic Variants	Number of Risk Loci Evaluated	Number of Total Risk Alleles Identified*	Polygenic Relative Risk**	Percentile Rank of Relative Risk**
Abdominal aortic aneurysm	6%	Unknown	3	3/6	1.0	40-50th
Atrial fibrillation	2%	10%	11	4/22	<0.6	0-10th
Coronary heart disease	6% (Age 40-59)	<10%	60	60/120	2.2	80-90th
Type 2 Diabetes	13% (Age 45-64)	5-10%	70	79/140	>3.6	90-100th
Hypertension	52%	<10%	3	2/6	1.0	40-50th
Obesity	37% (Age 40-59)	1-2%	7	12/14	1.2	60-70th
Platelet aggregation	Unknown	5-10%	4	0/8	<0.6	0-10th
QT prolongation	Unknown	7%	3	5/6	1.0	40-50th

*# of total possible risk alleles = # risk loci x 2 alleles per loci.

** As data utilized in this analysis were derived from non-longitudinal association studies, "Relative Risk from Common Genetic Variation" pertains to near-term risk of developing a phenotype (e.g. approximately 5 year risk), not lifetime risk. "Relative Risk from Common Genetic Variation" and "Percentile Rank of Relative Risk from Common Genetic Variation" values have been estimated using the 1000 Genomes European cohort.

CARDIAC RISK REPORT(CONTINUED)

It should be noted that this clinical report is limited to only a subset of variants that have met criteria for inclusion. Not all variants identified have been analyzed and not all regions included in the genome have been adequately sequenced. The polygenic predicted values for lipid levels are based on large epidemiologic studies and may not apply to each individual patient (model from N. Stitziel and S. Sunyaev, personal communication). The summary risk assessments above, for small-moderate effect alleles, are based on combining individual risk allele data in ways that may not always apply to each individual patient. These results should be interpreted in the context of the patient's medical evaluation, family history, and racial/ethnic background.

DETAILED VARIANT INFORMATION

MONOGENIC VARIANTS CONFERRING HIGH RISK OF DISEASE

This test did NOT identify individual genetic variants known or expected to cause existing cardiovascular disease or the development of cardiovascular disease in this individual's lifetime.

MONOGENIC VARIANTS POSSIBLY CONFERRING HIGH RISK OF DISEASE

Disease (Inheritance)	Gene (Transcript)	Variant (Classification)	Variant Frequency	Disease Prevalence	References
Brugada syndrome (Autosomal Dominant)	CACNA1C (NM_000719.6)	c.1468G>A p.Gly490Arg heterozygous (Uncertain significance)	5/4,020 European American	1-5/10,000	Antzelevitch 2007

VARIANT INTERPRETATION: The Gly490Arg variant in CACNA1C has been identified in a single patient with Brugada syndrome but was also identified in 5/4020 of European American chromosomes by the NHLBI Exome Sequencing Project (http://evs.gs.washington.edu/EVS/). Functional studies indicate this variant does not result in abnormal protein localization, but may lead to abnormal Ca2+ current (Antzelevitch 2007). Additional information is needed to fully assess the clinical significance of the Gly490Arg variant.

DISEASE INFORMATION: Brugada syndrome is characterized by cardiac conduction abnormalities (ST-segment abnormalities in leads V1-V3 on ECG and a high risk for ventricular arrhythmias) that can result in sudden death. Other conduction defects can include first-degree AV block, intraventricular conduction delay, right bundle branch block, and sick sinus syndrome. Adapted from GeneReviews abstract: http://www.ncbi.nlm.nih.gov/books/NBK1517/

FAMILIAL RISK: Brugada syndrome is inherited in an autosomal dominant manner. Most individuals diagnosed with Brugada syndrome have an affected parent. The proportion of cases caused by a de novo mutation is estimated at 1%. Each child of an individual with Brugada syndrome has a 50% chance of inheriting the mutation. Other biologically related family members may also have this variant.

METHODOLOGY

Genomic sequencing is performed using next generation sequencing on the Illumina HiSeq platform. Genomes are sequenced to at least 30X mean coverage and a minimum of 95% of bases are sequenced to at least 8X coverage. Paired-end 100bp reads are aligned to the NCBI reference sequence (GRCh37) using the Burrows-Wheeler Aligner (BWA), and variant calls are made using the Genomic Analysis Tool Kit. All variants in 103 monogenic cardiac disease genes are evaluated and classified according to pathogenicity. Pathogenic, likely pathogenic and variants of uncertain significance with higher suspicion for disease-causality are reported. In addition, risk alleles identified at 163 loci involved in cardiac disease are also reported. Odds ratios are combined to provide overall assessment of risk for broad phenotypes. It should be noted that this test does not sequence all bases in a human genome and will not detect or interpret all variants in a genome. Furthermore, the clinical significance of many variants is not well understood. The technical component of this test as developed and its performance characteristics determined by the Illumina CLIA Lab (San Diego, CA CLIA# 05D1092911) and the interpretive algorithms and clinical reports were generated by the Laboratory for Molecular Medicine at the Partners Healthcare Center for Personalized Genetic Medicine (LMM, 65 Landsdowne St, Cambridge, MA 02139; 617-768-8500; CLIA#22D1005307). This test has not been cleared or approved by the U.S Food and Drug Administration (FDA). The FDA has determined that such clearance or approval is not necessary.

LIMITATIONS

It should be noted that this test does not sequence all bases in a human genome and not all variants have been identified or interpreted.

CARDIAC RISK REPORT(CONTINUED)

Furthermore, not all cardiac disease-associated genes have been identified and the clinical significance of variation in many genes is not well understood. Therefore, it is recommended that genomic sequencing data is periodically reinterpreted, especially when new symptoms arise. It should be noted that the polygenic predicted values for lipid levels are based on large epidemiologic studies and may not apply to each individual patient (model from N. Stitziel and S. Sunyaev, personal communication). The summary risk assessments above, for small-moderate effect alleles, are based on combining individual risk allele data in ways that may not always apply to each individual patient.

REFERENCES

Antzelevitch C, Pollevick GD, Cordeiro JM, Casis O, Sanguinetti MC, Aizawa Y, Guerchicoff A, Pfeiffer R, Oliva A, Wollnik B, Gelber P, Bonaros EP, Burashnikov E, Wu Y, Sargent JD, Schickel S, Oberheiden R, Bhatia A, Hsu LF, Haïssaguerre M, Schimpf R, Borggrefe M, Wolpert C. 2007. Loss-of-function mutations in the cardiac calcium channel underlie a new clinical entity characterized by ST-segment elevation, short QT intervals, and sudden cardiac death. Circulation. 115(4):442-9.
